# Monitoring Fibrous Scaffold Guidance of Three-Dimensional Collagen Organisation Using Minimally-Invasive Second Harmonic Generation

**DOI:** 10.1371/journal.pone.0089761

**Published:** 2014-02-28

**Authors:** Robin M. Delaine-Smith, Nicola H. Green, Stephen J. Matcher, Sheila MacNeil, Gwendolen C. Reilly

**Affiliations:** 1 Kroto Research Institute, Department of Materials Science and Engineering, University of Sheffield, Sheffield, United Kingdom; 2 INSIGNEO Institute for in silico Medicine, Department of Materials Science and Engineering, University of Sheffield, Sheffield, United Kingdom; University of Minho, Portugal

## Abstract

The biological and mechanical function of connective tissues is largely determined by controlled cellular alignment and therefore it seems appropriate that tissue-engineered constructs should be architecturally similar to the *in vivo* tissue targeted for repair or replacement. Collagen organisation dictates the tensile properties of most tissues and so monitoring the deposition of cell-secreted collagen as the construct develops is essential for understanding tissue formation. In this study, electrospun fibres with a random or high degree of orientation, mimicking two types of tissue architecture found in the body, were used to culture human fibroblasts for controlling cell alignment. The minimally-invasive technique of second harmonic generation was used with the aim of monitoring and profiling the deposition and organisation of collagen at different construct depths over time while construct mechanical properties were also determined over the culture period. It was seen that scaffold fibre organisation affected cell migration and orientation up to 21 days which in turn had an effect on collagen organisation. Collagen in random fibrous constructs was deposited in alternating configurations at different depths however a high degree of organisation was observed throughout aligned fibrous constructs orientated in the scaffold fibre direction. Three-dimensional second harmonic generation images showed that deposited collagen was more uniformly distributed in random constructs but aligned constructs were more organised and had higher intensities. The tensile properties of all constructs increased with increasing collagen deposition and were ultimately dictated by collagen organisation. This study highlights the importance of scaffold architecture for controlling the development of well-organised tissue engineered constructs and the usefulness of second harmonic generation imaging for monitoring collagen maturation in a minimally invasive manner.

## Introduction

The micro-architecture of human connective tissues is dictated by controlled cellular alignment, which determines the biological and mechanical function of the tissue. In order to replicate this, it is important that tissue engineered constructs mimic the architecture of the target tissue in the body. For example, tendon tissue is composed of highly aligned arrays of dense collagen fibres (dense regular) with tendon fibroblasts elongated in-between and along the length of the fibres. This allows for strong tensile properties in the direction parallel to the fibres. In the dermal region of skin, fine and dense woven bundles of collagen and elastic fibres (dense irregular) are present giving skin good strength and elasticity in multiple directions.

Tissue-engineered constructs typically consist of cells seeded into synthetic or naturally-derived scaffolds that may be in the form of a hydrogel or a porous scaffold or foam with the aim of forming a 3D network of cells and matrix [1]. However, these scaffold types do not precisely control cell shape and orientation, which ultimately results in poor ECM organisation compared with native tissue. Scaffold fabrication techniques such as salt leaching and lyophilisation often generate pore sizes that are of the order of hundreds of microns [2], [3], and this results in cells stretching out along the pore wall as if it was a flat or slightly curved surface. While voids with a size larger than that of the cell allow for sufficient nutrient diffusion and space for matrix deposition, they also make it difficult for cells to bridge the pores. In the body, most cells have multiple attachments to different points within a 3D architecture, which enables them to migrate and proliferate as well as facilitating controlled secretion of matrix.

There are a number of techniques that have been developed with the aim of controlling cellular behaviour on both the micro- and nano-scale [4] by controlling the cell environment. There are numerous examples where defined micro- and nano-architectures have been generated by chemical or topographic patterning on two-dimensional (2D) surfaces to control cellular alignment and behaviour [5], [6], however these do not reflect the three-dimensional (3D) fibrous nature of most tissues in the body and translating these technologies into 3D scaffolds remains a major challenge.

In order to control cell orientation and subsequent matrix organisation, the use of a construct with a controllable architecture that matches the relevant anatomical micro-architecture would be ideal. Electrospinning is a straightforward and versatile technique that offers itself to a wide range of polymers and composite materials capable of producing continuous fibres with typical diameters in the range of a few micrometers down to tens of nanometers with high surface area to volume ratios [7]. Electrospun mats can be produced that contain randomly orientated, non-woven fibres or substrates with highly aligned fibres [8]. Polycaprolactone (PCL) is a biodegradable aliphatic polyester that can be electrospun easily and has found wide use in tissue engineering strategies.

Collagen is the major structural protein in connective tissues, comprising 56–70% of skin and 75–85% of tendon (dry weight) [9], and its organisation ultimately determines the mechanical properties of the native tissue. Therefore, long-term monitoring of collagen deposition by cells in culture is of great importance and this ideally requires a technique that is minimally- or non-invasive. Most techniques used for observing collagen organisation are invasive, require numerous preparation steps (which often alter collagen structure), and result in the destruction of the sample. Second harmonic generation (SHG) is a minimally-invasive, two-photon based technique that allows for label-free imaging of non-centrosymmetric molecules. Collagen is a strong source of SHG [10] and the intensity of the signal produced depends on a number of factors including the amount of collagen deposited, collagen type and degree of microstructural order [11], [12]. The sensitivity to directional order on sub-diffraction-limited scales separates SHG from assays that primarily measure collagen abundance, such as picrosirius-red staining or fluorescent microscopy. As the technique requires no sample preparation, collagen is imaged in its secreted form and this can be observed in a rapid manner at different depths within the construct. The intrinsic optical sectioning effect of two-photon microscopy naturally provides 3D spatially resolved information with sub-micron resolution.

It has been shown previously that scaffold fibre orientation can control fibroblast morphology [13], [14] and cell orientation controls the alignment of cell-secreted collagen [15]. When human ligament fibroblasts (HLFs) were seeded on aligned nanofibres, they adopted an elongated spindle-shape orientated in the fibre direction [16]. After 7 days, significantly more collagen was also synthesised on aligned nanofibers compared with randomly orientated ones, which was also organised in the fibre direction. This suggests that cellular morphology is a major contributing factor as to how ECM is produced and organised, and that matrix orientation could be controlled if the orientation of the cell was controlled. However, there are few studies observing longer term culture of cells (>14 days) on orientated substrates and little information on how collagen is deposited and organised throughout the construct over the culture period.

The main aim of the current study was to further investigate how scaffold fibre orientation influences new matrix production and distribution, and what effect matrix organisation has on construct mechanical properties. Specifically, this was achieved by comparing how randomly orientated and highly aligned electrospun micro-fibres influenced fibroblast migration and proliferation up to 21 days in culture and what affect this had on subsequent collagen secretion and organisation. A main objective was to evaluate the usefulness of SHG to monitor the production and organisation of cell-deposited collagen in its secreted form at different scaffold depths over the culture period. SHG proved to be an informative minimally-invasive technique for monitoring the 3D organisation of cell-secreted collagen which was directed by scaffold fibre orientation and this ultimately dictated construct tensile properties.

## Materials and Methods

### Ethics Statement

Collection of human skin was performed on an anonymous basis under a Human Tissue Authority (HTA) Research Tissue Bank Licence (No. 12179). Patients undergoing elective surgical procedures (abdominoplasties and breast reductions) gave informed written consent for skin not required for their treatment to be used for research purposes.

### Cell culture

Normal human dermal fibroblasts (HDFs) were isolated from skin obtained from consenting patients undergoing elective surgical procedures as described previously [17]. Cells were isolated and expanded in complete cell culture media consisting of Dulbecco's Modified Eagles's Medium (DMEM) (Biosera, UK) supplemented with 10% foetal calf serum, 2 mM L-glutamine, and 100 µg/ml penicillin/streptomycin. For experiments, HDFs were used between passages 3–7 with the addition of ascorbic acid-2-phosphate (50 µg/ml) to the culture media after 24 hours of seeding. HDFs were incubated at 37°C in the presence of 5% CO_2_ with fresh media changes every 2–3 days. All reagents were obtained from Sigma-Aldrich (UK) unless otherwise stated.

### Fabrication of electrospun PCL micro-fibres

Solutions of poly(ε-caprolactone) (PCL) (Mn 80 kDa) (Sigma-Aldrich, UK), a bioresorbable aliphatic polyester that has shown excellent biocompatability with mesenchymal cells [18], were prepared at a concentration of 15 w% in dichloromethane and stirred at room temperature for 48 h to ensure complete dissolution and solution homogeneity. Solutions were stored sealed at room temperature for up to one week. Electrospinning was performed at room temperature and fibres were collected using a horizontal variable speed steel rotating drum collector (diameter 6 cm) covered with aluminium foil. Solutions were poured into 1 ml hypodermic syringes with a 2 mm diameter blunt tip needle connected to a variable high voltage power supply (0–30 kV) and dispensed using programmable micro-syringe pump. For both fibre orientations, solution flow rate was set at 4 ml/h, the voltage was set at 11 kV, and the distance between the collector and needle tip (working distance) was 20 cm. For randomly orientated fibres, the collector speed was 200 rpm and for highly aligned fibres the collector speed was 2000 rpm. After spinning, fibrous scaffolds were placed under vacuum at room temperature for 24 h and then stored at 4°C in sealed plastic bags for up to 6 months.

### Physical characterisation of electrospun fibres

Electrospun fibre diameters and fibre orientations were determined manually from SEM micrographs using Analysis in Java (ImageJ, National Institutes of Health). Briefly, the correct scale was set and lines were drawn across fibres (edge to edge) and the distance recorded. For orientation analysis, lines were drawn along the fibre length and the deviation angle from a manually assigned line of orientation was measured. For water contact angle measurement, scaffolds were cut into 10 mm diameter circular discs and a small drop of ultrapure H_2_O was dispensed onto the scaffold using a micro-needle and the contact angle was measured using a Goniometer (ramé-hart instrument co., USA). Scaffold porosity was determined firstly by calculating scaffold density whereby 13 mm scaffold circles were measured for thickness and weighed. Scaffold porosity (ε) was then calculated using the following formula (where ρ =  density of scaffold and ρ_0_  =  density of the bulk material):
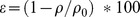



### Fibroblast culture on electrospun fibres

Scaffolds were cut to size and sterilised using 0.1% peracetic acid at room temperature for 3 h. Samples were rinsed and wetted thoroughly with PBS then left to soak in complete DMEM overnight before seeding the following day. PCL fibres were seeded with 100,000 HDFs using marine-grade steel seeding rings (internal Ø 10 mm) and incubated in complete DMEM in 6-well plates for 24 hours before transferring scaffolds to new well plates and adding fresh medium. For studies involving quantification of total DNA and collagen, and visualisation of collagen and cell morphology, scaffolds were cut into circles (Ø = 13 mm) with a thickness of 300 µm. For migration studies, scaffolds were cut into larger circles (Ø = 30 mm) and for mechanical testing, scaffolds were cut into rectangular strips (30×10 mm) with a thickness of between 100–150 µm. Thickness measurements were made using a digital micrometer accurate to ±1 µm by placing scaffolds in between glass cover slips and subtracting the thickness of the glass cover slip.

### Cell migration and metabolic activity

Assessment of cell migration and metabolic activity was determined at days 3, 7 and 12 using the MTT (A3-(4,5-dimethylthiazol-2-yl)-2,5-diphenyltetrazolium bromide) assay (Sigma, UK). Briefly, cells were washed free of media with PBS and incubated with MTT solution for 40 min at 37°C. Photographic images of the resulting purple formazan salt were captured and migration distance was determined from images using Image J plotted as the distance from the centre of the cell-seeded ring to the MTT stain that was furthest away. Cell metabolic activity was quantified by destaining the purple formazan product with acidified isopropanol, and recording the absorption at 562 nm.

### Cell morphology

Cellular morphology was visualised at days 7 and/or 21 using fluorescence microscopy. DAPI (4′,6-Diamidino-2-phenylindole dihydrochloride) (1 µg/ml) and Phalloidin-TRITC (Phalloidin-Tetramethylrhodamine B isothiocyanate) (1 µg/ml) (Sigma, UK) staining were used for cell nucleus and actin-cytoskeleton respectively. Briefly, cells were washed with PBS and fixed with 4% formaldehyde for 20 min followed by treatment with 0.5% Triton-X for 5 min to permeabilize the cell membrane. Images were captured using a Zeiss LSM 510 Meta upright laser-scanning confocal microscope (Carl Zeiss MicroImaging, Germany) using a 40x 1.3 NA oil immersion objective at a range of depths by moving the focal plane towards the top of the scaffold until no cells were visible and then moving the focal plane down at set increments. DAPI was excited using an 800 nm two-photon laser and emission was detected between 435 and 485 nm. TRITC was excited with a 543 nm laser and emission was detected between 565 and 615 nm.

### Total DNA and collagen quantification

Total DNA was measured at days 7, 14 and 21 of culture using a fluorescent Quant-iT PicoGreen dsDNA reagent assay kit (Invitrogen, UK). Briefly, cells on scaffolds were washed free of media with PBS and placed into a micro-tube containing a known volume of a cell lysis buffer solution (10 mM Tris +1 mM MgCl_2_ +1 mM ZnCl_2_ + 0.1% Triton-X) followed by 1 min vortexing and centrifugation at 10,000 rpm for 5 min. Samples were then stored at 4°C for 24 h. Samples were freeze-thawed 3 times before a known volume of cell lysate was added to the provided Tris-buffered EDTA solution. The Quant-iT PicoGreen reagent was then added, which binds to the double-stranded DNA in solution, and fluorescence intensity was recorded using a FLx800 microplate fluorescence reader (BioTek, UK) using 485 nm excitation and 520 nm emission. Total DNA was converted to ng DNA/sample from a standard curve.

Total cellular collagen production was quantified at days 7, 14 and 21 by staining with a 0.1% Picrosirius red (SR) solution (0.1% Direct Red 80 in saturated picric acid) (Sigma, UK) for 18 hours on a platform shaker. The remaining SR solution was washed away with deionised water and the resulting stain was removed with methanol:0.2 M sodium hydroxide (1∶1) for 30 minutes on a platform shaker. The absorbance of the resulting solution was then measured at 490 nm on a 96-well plate reader. Total collagen was also normalised to ng DNA.

### Second harmonic generation and collagen visualisation

Cell-deposited collagen was visualised in TE constucts at days 14 and/or 21 from SHG images obtained at varying scaffold depths using a Zeiss LSM 510 Meta upright laser-scanning confocal microscope attached to a tuneable (700–1060 nm) Chameleon Ti:sapphire multiphoton laser (Coherent, CA, USA). For imaging scaffold fibre and collagen proximity in unfixed or formalin-fixed constructs, the multiphoton illumination wavelength (λ_I_) was set at 800 nm and SHG emissions collected in a 10 nm bandpass filter centred around 400 nm. For studies monitoring collagen deposition and organisation over time and 3D image construction of unfixed constructs, samples were illuminated at 940 nm and SHG emissions were collected in a 10 nm bandpass filter centred around 474 nm. For imaging of cell-free PCL fibres and fresh rat tail tendon (kindly provided by Juliet Bell), samples were illuminated at both 800 nm and 940 nm. All imaging was performed using a 40x 1.3 NA oil immersion objective with the pinhole set to maxium using a laser excitation power of 20 mW and SHG was collected in the backward direction after filtration through a primary dichroic (HFT KP650). All other imaging parameters were optimised (for example detector gain and scan speeds) and conditions were kept constant for all samples at each experimental time point, unless stated otherwise. Fluorescent imaging of Sirius red stained constructs was performed on the same confocal system at day 21 using a 543 nm excitation laser with emission detected between 565 and 615 nm.

### Mechanical testing

Mechanical testing of dry and wet PCL fibres or cell-seeded constructs was performed in a biodynamic chamber filled without (dry) or with (wet) complete medium using a BOSE ELF 3200 equipped with a 22.2 N load cell (ElectroForce Systems Group, BOSE, Minnesota, USA). Separate cell-seeded constructs were tested at day 0 (no cells), and days 2, 7, 14, and 21 (with cells). The gauge distance was set at 10 mm and samples were strained at a rate of 0.1 mm/s to a maximum strain distance of 6 mm and the resulting force applied to the scaffold/construct was recorded along with the movement of the cross head. Recorded data was used to generate stress/strain curves and Young's modulus of elasticity was calculated from the gradient of the linear (elastic) region of the slope.

### Statistical analysis

All experiments were performed at least twice with triplicate samples for each assay unless stated otherwise. Collagen visualisation using SHG was performed on one sample of each fibre orientation per experimental run with images being obtained from the central region of the scaffold. Cells cultured on random and aligned scaffolds were compared for statistical differences using an unpaired Student's t-test. For comparisons of more than two sample means, one-way ANOVA followed by Tukey's post-hoc test was performed. All graphs are mean ± SD and significant differences are marked when *p<0.05*.

## Results

### Electrospun PCL fibres

**Figure 1:**
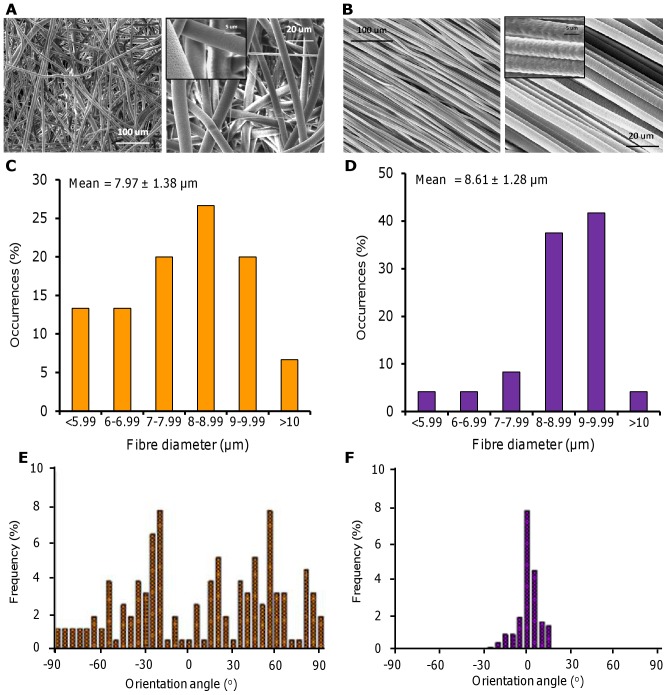
Electrospun PCL fibres with a random orientation or highly aligned. Random (A) and aligned (B) fibres were formed from a PCL (80 kDa) 15 w% in DCM solution. Conditions were 4 ml/h, 11 kV, 20 cm Wd, 200 rpm (A) and 2000 rpm (B). SEM micrographs (A–B) show different magnifications (insert scale bar is 5 µm). Fibre diameter distribution (C–D) and frequency of fibre orientation (E–F) were calculated from 150 fibres (n = 3).

Electrospun scaffolds were fabricated with two distinct fibre orientations; either randomly arranged (Fig. 1A) or highly aligned (Fig. 1B). Both scaffolds contained fibres with a similar mean diameter and fibre distribution (Fig. 1C–D) but with differing degrees of orientation (Fig. 1E–F). Scaffold porosity was greater than 75% for both fibre configurations with random fibrous scaffolds being more porous than aligned fibrous scaffolds. Random fibrous scaffolds showed isotropic mechanical properties while aligned fibrous scaffolds were anisotropic and were nearly 1000 times stiffer when strained parallel to the fibre direction compared with perpendicular strain (Table 1).

**Table 1:** Physical and mechanical properties of random and aligned electrospun PCL fibres.

Scaffold	Porosity (%)	Water contact angle (^o^)	Young's Modulus (Mpa)	TS at 100% strain (MPa)
**Random**	85.5±1.8*	135±3*	3.31±0.37*	0.59±0.07*
**Aligned**	77.3±1.1	64±4 (Parallel) 118±17 (Across)	32.4±5.8 (Parallel) 0.037±0.01 (Across)	4.58±0.63 (Parallel) 0.017±0.004 (Across)

Parallel refers to values obtained from aligned fibres in the longitudinal direction and across refers to values obtained from aligned fibres perpendicular to the fibre direction. Values are quoted as mean ± SD (n = 6), *p<0.05 for random versus both aligned orientations.

### Cell metabolic activity and migration

**Figure 2:**
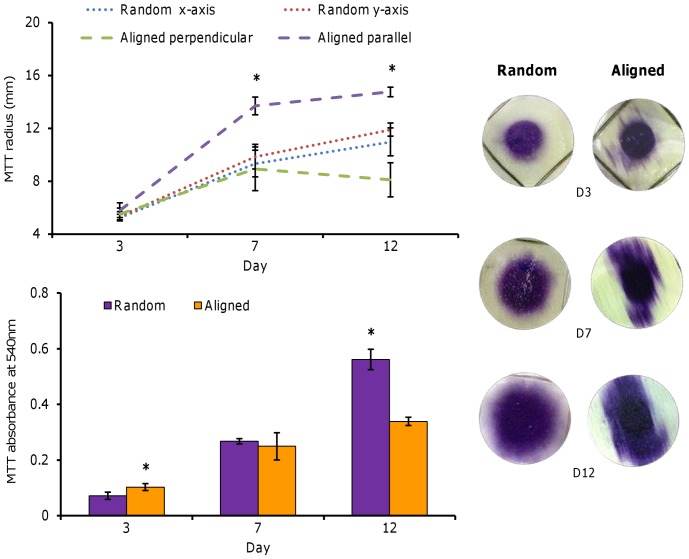
HDF viability and migration on PCL fibres. HDFs seeded into the centre of random and aligned fibres and assayed with MTT at days 3, 7 and 12 to observe cell migration and viability. Cells on random fibres migrated equally in all directions whereas cells on aligned fibres migrated along the direction of the fibre. Data is mean ± SD (n = 6), *p<0.05.

HDF metabolic activity increased with culture time on both scaffold fibre orientations suggesting that cells proliferated over the course of the culture period (Fig. 2). At day 3, metabolic activity was significantly highest on aligned scaffolds (*p<0.05*) but at day 12, it was almost 2-fold greater on randomly orientated scaffolds compared with aligned fibres. Migration of HDFs was inferred from images obtained of the soluble purple product formed by the MTT (Fig. 2). At day 3, both scaffolds showed a similar circular MTT stain from where the cells were seeded in the metal ring but some cells appeared to have partially migrated along the fibre direction. At day 7, cells on random scaffolds had begun to migrate evenly in all directions increasing the diameter of the stained area almost 2-fold whereas cells on aligned scaffolds had migrated along the fibre direction and reached the edges of the scaffold. By day 12, cells on aligned scaffolds had covered the scaffold from end to end in the direction of the fibre orientation, whereas cells on the random fibres continued to spread evenly away from the centre of the scaffold.

### Total DNA and Sirius red staining

**Figure 3:**
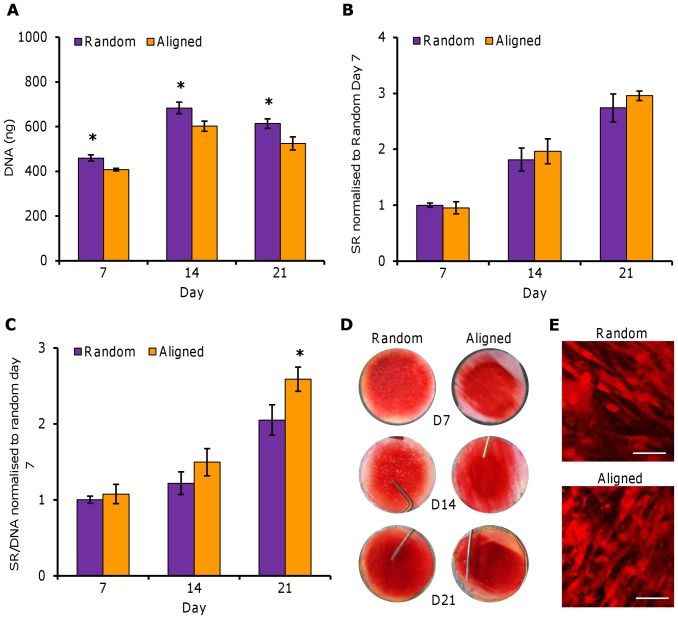
Total DNA and SR staining of HDFs on PCL fibres. HDFs seeded on random and aligned electrospun PCL fibres were assayed for total DNA by Pico Green and collagen deposition by SR staining. Total DNA (A) and total SR stain (B) was quantified at days 7, 14 and 21. SR was normalised to total DNA (C) at each time point. SR staining can be visualised (D). Data is mean ± SD (n = 6–9), *p<0.05.

Total DNA increased in both scaffold orientations from day 7 to 14 and then reduced slightly at day 21 (Fig. 3A). At all time points, DNA levels were significantly higher (*p<0.05*) in random scaffolds. Total collagen was assayed at days 7, 14 and 21 and the amount of collagen deposited increased in both scaffolds in a linear fashion, at the same rate, over the course of the culture period (Fig. 3B). Normalising total collagen to total DNA showed that cells on aligned scaffolds produced more collagen per cell at day 14 and significantly higher levels at day 21 (*p<0.05*) (Fig. 3C). SR staining was seen to cover the scaffold evenly by day 21 on random fibres but only covered the scaffold from end to end in the fibre direction on aligned fibres (Fig. 3D). Closer inspection of the resulting stain using confocal microscopy showed that collagen on aligned constructs was generally orientated in the direction of scaffold fibre alignment (Fig. 3E).

### Cell Morphology

**Figure 4:**
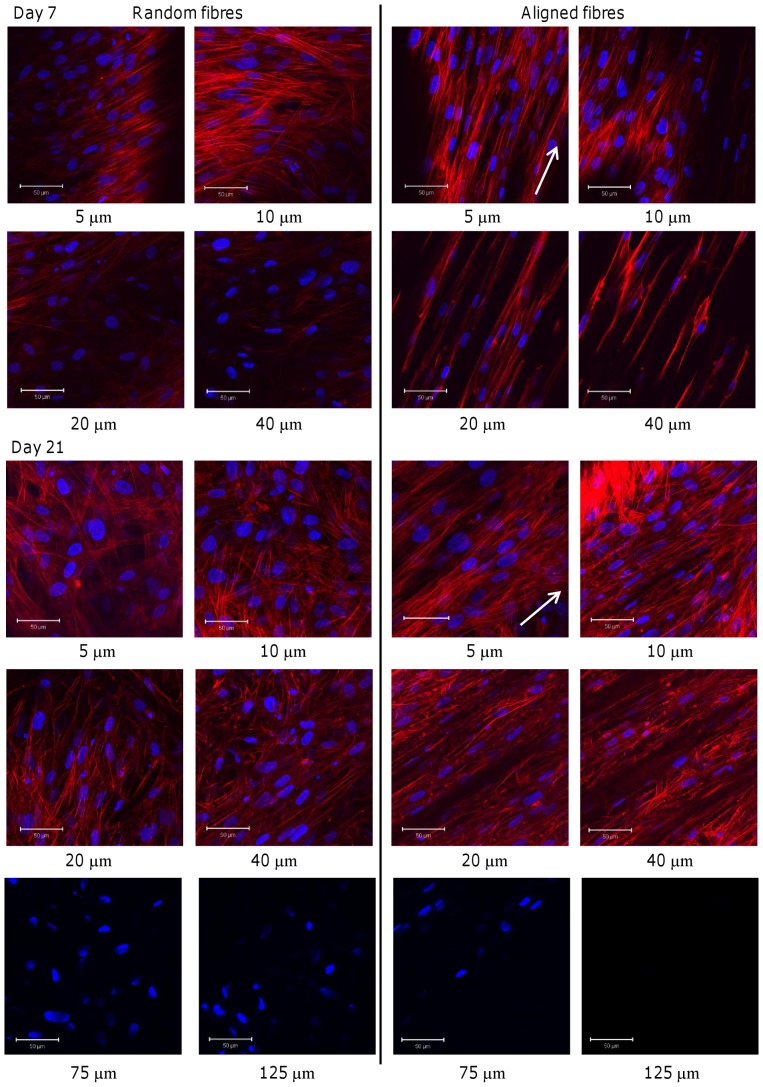
HDF morphology on PCL fibres. Nucleus (DAPI in blue) and cytoskeleton (phalloidin-TRITC in red) of HDFs seeded on random and aligned scaffolds. HDF morphology was visualised at day 7 (top) and 21 (bottom) imaged at different depths (5–40 µm). Cells on random fibres were generally attached in multiple directions while cells on aligned fibres were spindle shaped and aligned in the fibre direction. White arrows indicate direction of scaffold fibre alignment. Scale bars are 50 µm.

At day 7, cells seeded on random fibres showed different morphologies depending on the depth at which they were found in the scaffold (Fig. 4). Near the top surface of the scaffold (5 µm), cells were spindle shaped and tightly packed, and the direction of cell orientation changed across the scaffold (not shown). At 10 µm deep, cells adopted a more star-like shape as some cells appeared to attach to multiple fibres causing them to branch out. At 20–40 µm deep, most of the cells were attached to multiple fibres and spread out in a star shape with the number of cells decreasing with increasing depth. On aligned fibrous mats, cells were also spindle shaped on the surface (5 µm) but were more elongated than cells on random mats and also spread along the direction of fibre alignment. This was also observable at 10–40 µm deep, but at 20–40 µm HDFs also appeared to have attached along individual fibres and elongated further still. Cells imaged at day 21 (Fig. 4) on the random scaffolds showed a similar trend in cell morphology at all depths compared with day 7 but more cells were now observable with increasing depth. On the aligned fibres, while most cells were still orientated in the fibre direction at depths of 5–20 µm, there were also a number of cells that no longer aligned along the fibre direction suggesting that cellular orientation was being lost over time. Both constructs were also imaged for cell nuclei at 75–125 µm with fewer visible cells in aligned constructs at 75 µm and no visible cells at 125 µm while cell nuclei were observed at both depths in random constructs.

### Second harmonic generation visualisation of scaffolds and collagen

Preliminary work showed that PCL scaffold fibres produced SHG when illuminated at 800 nm but this signal was absent when 940 nm light was used, while SHG from fibrous collagenous tissue (rat tail tendon) could be visualised using illumination at both 800 nm and 940 nm (Fig. S1). This discrepancy was utilised to image unfixed and fixed constructs at 800 nm to identify the proximity of cells and secreted collagen to scaffold fibres, while 940 nm was used to clearly monitor collagen deposition and organisation in unfixed constructs over time.

**Figure 5:**
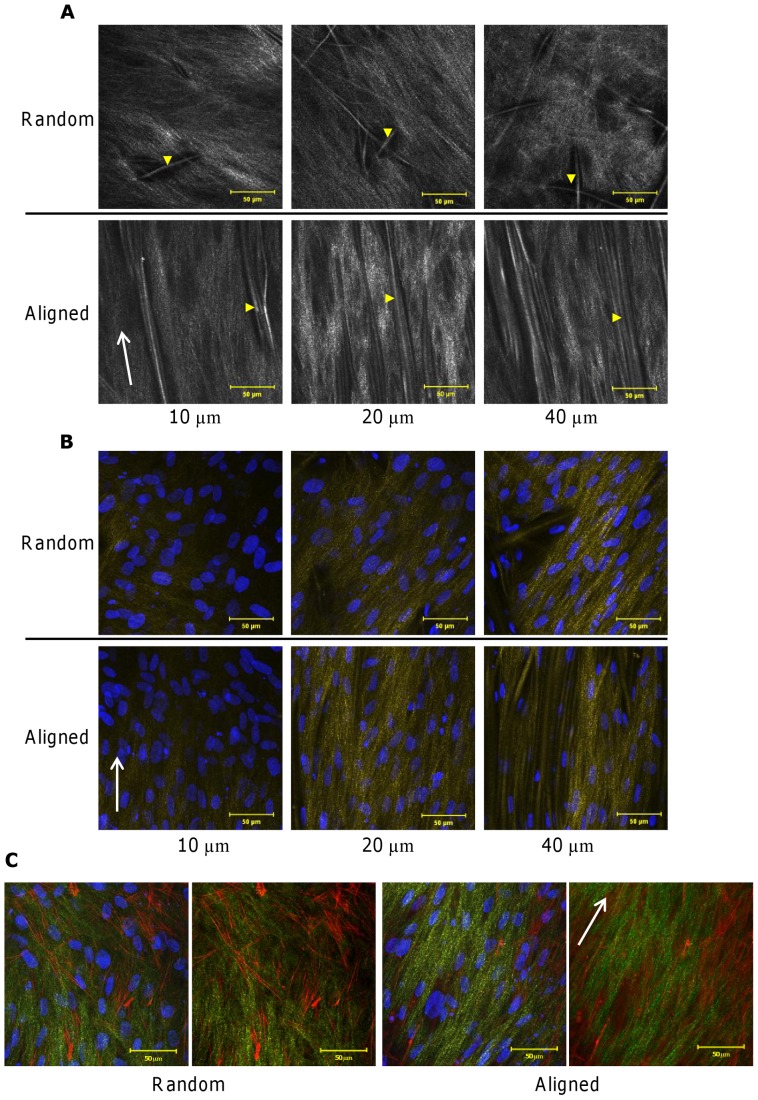
Fibroblasts and secreted collagen grow into constructs and align with scaffold fibre orientation. Fibroblast-secreted collagen and scaffold fibres were visualised using SHG emissions collected at depths of 10–40 µm (A). Yellow arrow heads indentify PCL fibres of the scaffold. Fibroblast-seeded constructs were fixed and visualised via SHG emissions (collagen and scaffold fibres in yellow) and DAPI staining (cell nucleus in blue) at depths of 10–40 µm (B). Fixed constructs were also visualised for fibroblast cytoskeleton (phalloidin-TRITC in red) proximity to secreted collagen (SHG in green) (C). SHG was obtained using 800 nm illumination and all images were collected after 21 days of culture. White arrows indicate scaffold fibre orientation.

At day 21, unfixed constructs were imaged at 3 depths (10, 20 and 40 µm) and SHG emanating from both cell-secreted collagen and scaffold fibres was observed (Fig. 5A). Collagen was deposited in the scaffold between the scaffold fibres of both constructs and the number of visible scaffold fibres increased deeper into the construct. In aligned constructs, collagen appeared more organised and orientated while collagen deposited in random constructs did not favour a particular orientation. Next, the relationship of fibroblasts to scaffold fibres and cell-secreted collagen was investigated in formalin-fixed constructs. Note that SHG emissions were less intense in fixed samples compared with unfixed samples. Cell nuclei were observed to elongate and orientate along the length of the scaffold fibres and in the direction of secreted collagen in aligned constructs at all imaged depths (Fig. 5B). In random constructs, cell nuclei did not adopt a preferential orientation at any depth and when nuclei appeared to have an aspect ratio closer to 1, surrounding collagen appeared to become more disorganised compared to cells that were more elongated (aspect ratio greater than 1). Cytoskeletal staining of fibroblasts showed that elongation of the cell body was accompanied by a more organised, aligned collagen matrix where as a more evenly spread cell, as seen on the random constructs, resulted in a more disorganised collagen matrix (Fig. 5C).

**Figure 6:**
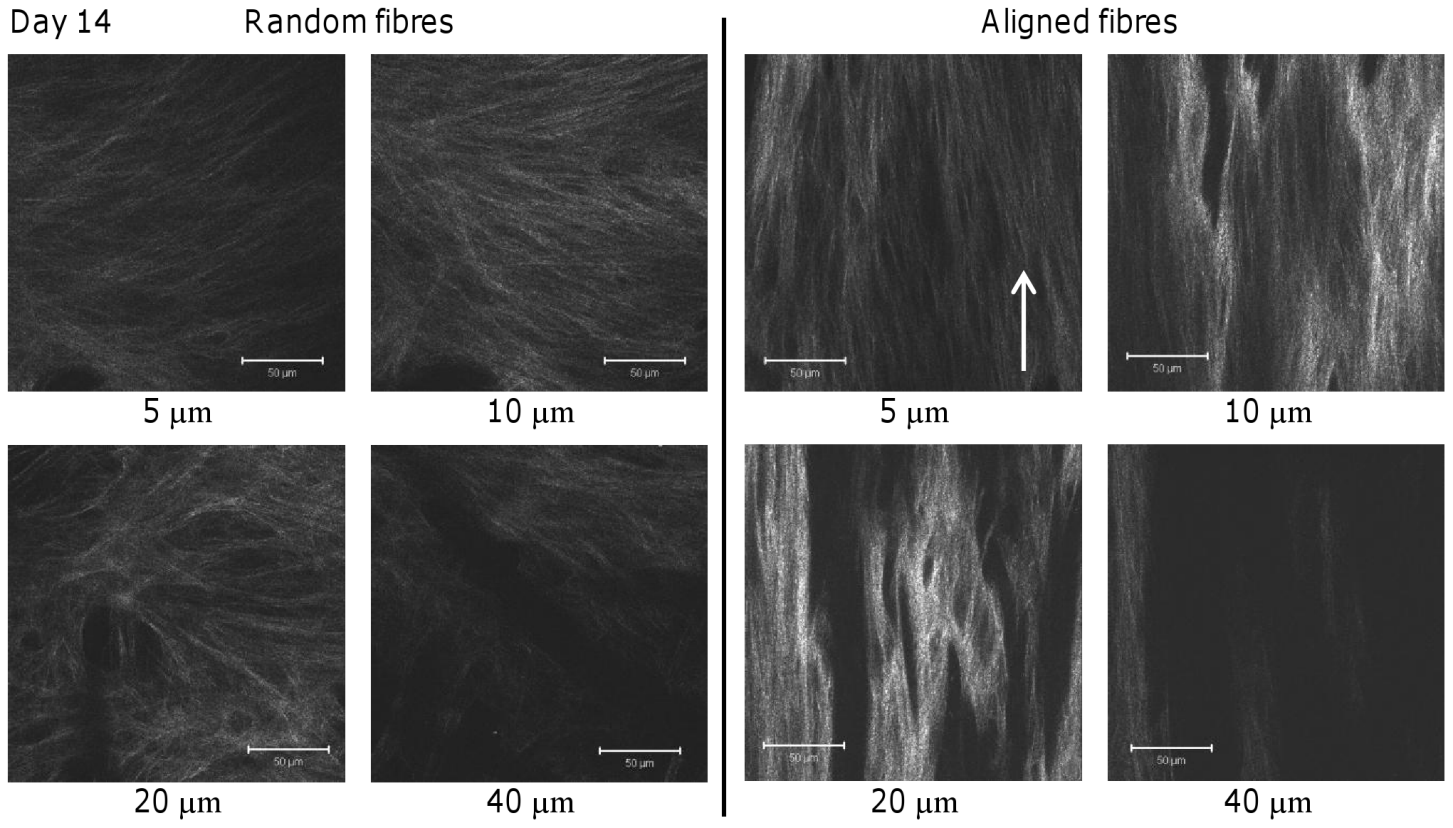
Collagen SHG from HDF-seeded constructs at day 14. SHG images of collagen matrix deposited on random and aligned scaffolds were obtained at day 14 taken from different depths. An increase in SHG intensity suggests an increase in collagen production and/or a more organised collagen fibrous network. Arrow indicates direction of scaffold fibre alignment. Scale bars are 50 µm.

**Figure 7:**
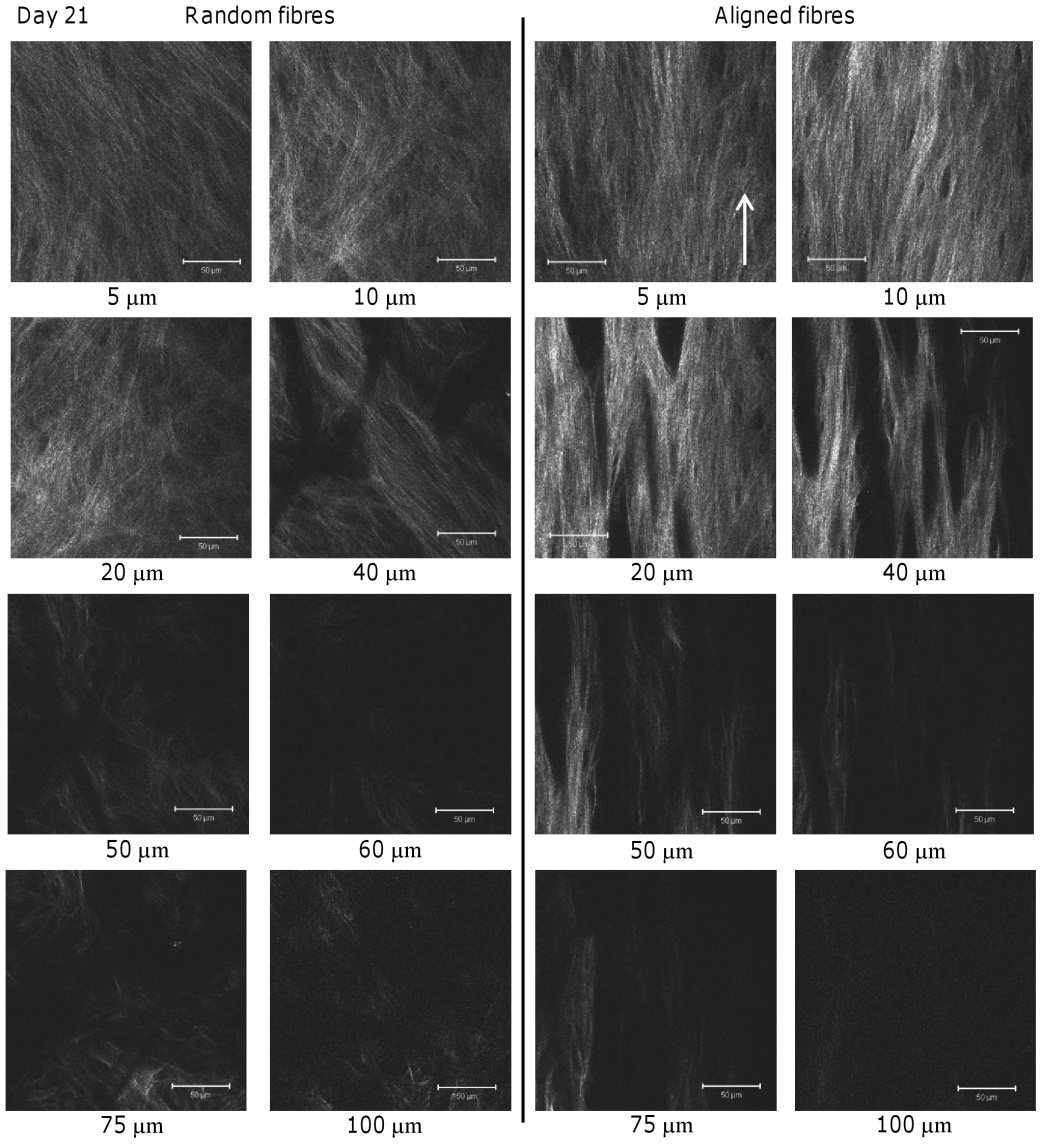
Collagen SHG of HDF-seeded constructs at day 21. SHG images of collagen matrix deposited on random and aligned scaffolds were obtained at day 21 at different depths. SHG intensity and area coverage is greater at all depths than the equivalent depths at day 14 suggesting increased collagen deposition and organisation. For images taken at 75–100 µm on both scaffolds, laser power was set to maximum in order to see any SHG present. Arrow indicates direction of scaffold fibre alignment. Scale bars are 50 µm.

SHG was obtained from unfixed cell-secreted collagen at different depths on both scaffold orientations at days 14 (Fig. 6) and 21 (Fig. 7). At day 14, SHG from random scaffolds showed a fibrous matrix with no preferential orientation at all depths (Fig. 6) and at 20–40 µm deep, the collagen fibres appeared spread in all directions. On aligned fibres, collagen SHG showed matrix orientation and alignment at all depths. The SHG signal was more intense at all depths on aligned scaffolds, compared to random scaffolds, and this was most evident at 10–20 µm, although random fibres appeared to show more area coverage.

At day 21, signal intensity and area coverage had increased on both scaffold fibre types at all depths indicating continued collagen deposition and/or organisation over the culture period (Fig. 7). As seen at day 14, at all depths the collagen matrix appeared randomly orientated on random fibres but orientated on aligned scaffolds. The greatest intensities for both scaffolds were at the depths of 10–20 µm, and at this time point there was a large increase in SHG intensity at 40 µm deep for both scaffolds. SHG was also obtained up to 100 µm into the scaffolds and images showed the same trends as before but at 100 µm there appeared to be more collagen present in the random scaffold than the aligned scaffold.

**Figure 8:**
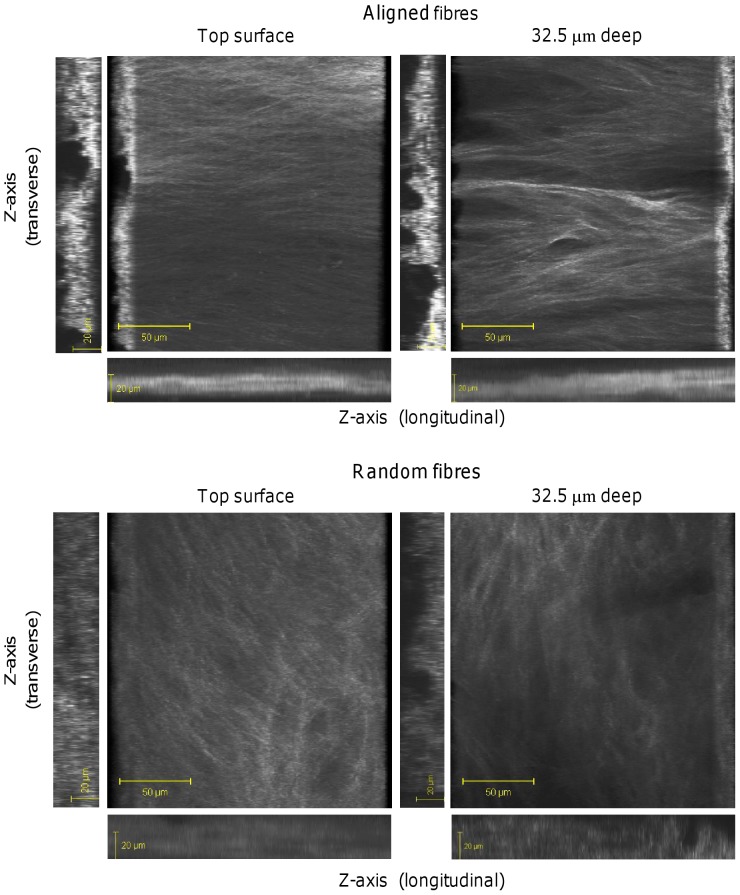
3D images of collagen SHG from HDF-seeded constructs at day 21. Images of HDF deposited-collagen on random and aligned scaffolds were compiled from 35 µm z-stacks (2.5 µm slices). A rotated 3D view from the top and bottom of the constructs are shown along with 2D images in the z-plane taken from both the horizontal and longitudinal edges.

In order to get a true sense of the 3D collagen organisation in the fibrous constructs, individual images were obtained from each construct in the z-plane (2.5 µm slices) at day 21 and then compiled together into 3D images (Fig. 8). As seen with the previous SHG images (Fig. 6–7), deposited collagen fibres were highly organised throughout the construct on aligned scaffolds and the signal was more intense. Images constructed in the z-plane showed more intense SHG for the cross section (x-z plane) compared with the longitudinal direction (y-z plane) for both constructs, while both images were more intense for aligned constructs compared with random constructs. However, collagen was deposited uniformly throughout the whole of the random construct, which was not the case for aligned constructs.

### Tensile properties of cell-seeded fibres

**Figure 9:**
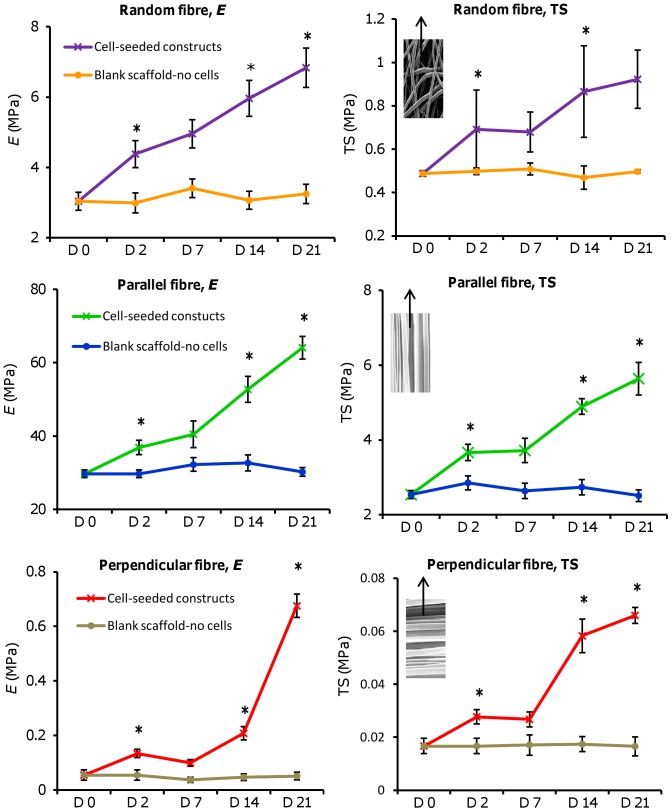
Tensile testing performed on blank and HDF-seeded constructs of different fibre orientations. Scaffold properties were observed at day 0 (blank-no cells) and days 2, 7, 14, and 21 (with cells). Notice the difference in the y-axis scale for each scaffold orientation (parallel > random > perpendicular). SEM images indicate scaffold orientation with arrow showing direction of tensile force applied. Data is mean ± SD (n = 6: random and parallel or n = 3: perpendicular), *p<0.05 verses previous time point tested.

In order to test the effect that cell-deposited matrix had on construct tensile properties, cell-free (blank) and cell-seeded scaffolds were tested for Young's modulus of elasticity (*E*) and the tensile strength at 50% strain (TS) across the culture period (Fig. 9).

At all time points, *E* and TS of cell-free scaffolds did not differ significantly for each fibre orientation compared with day 0 and maintained constant properties. Tensile properties were highest for aligned constructs strained parallel to the fibre direction for all time points tested, followed by random constructs, and then aligned constructs strained perpendicular to the fibre direction. All three scaffold orientations seeded with cells had significantly higher *E* and TS at all time points compared with scaffolds with no cells (*p<0.05*), indicating that the cells and deposited matrix increased scaffold strength. Both *E* and TS increased with time in culture on all cell seeded scaffolds with the exception of days 2–7 which had similar properties for all scaffolds. For perpendicularly strained scaffolds there was a dramatic increase in *E* from days 14 to 21 and a similar trend in the TS from days 7–14. For randomly orientated scaffolds, *E* increased from 3 MPa (no cells) to 7 MPa (day 21 with cells), whereas *E* of parallel strained scaffolds showed a larger increase from 30 MPa (no cells) to >60 MPa (day 21 with cells).

## Discussion

In order to develop tissue engineered constructs that are suitable for implantation, it is important that physiological function is replicated as well as tissue mechanical properties. To achieve this, scaffold materials should replicate the structural architecture of the native ECM with the ability to control cell behaviour and differentiation. Electrospun scaffolds composed of randomly orientated or highly aligned fibres replicate two major types of tissue organisation found within the body; Aligned fibres replicate the high degree of organisation found in ligament/tendon and muscle [16], [19] and random fibres represent the dense irregular organisation found in skin or cornea [7], [20].

This study showed that electrospun PCL micro-fibres influence fibroblast behaviour over 21 days of culture and that scaffold fibre orientation dictates how cell-secreted collagen is deposited throughout the construct. The minimally-invasive technique of SHG allowed for the visualisation of fibrous collagen in its unaltered secreted form, showing increased deposition and construct infiltration with time. Depth profiling of the constructs with SHG also showed that collagen deposition and/or organisation varies at different depths within the construct, dependant on the initial scaffold architecture. Collagen deposition also appeared to directly influence construct mechanical properties.

Cell metabolic activity was initially highest on aligned scaffolds (D3) but at the end of culture was highest on random scaffolds (D12). Initially, cells migrate fast along the aligned fibres but once they reach the end of the scaffold there is limited space for proliferation. In addition cells migrated deeper into random fibrous scaffolds than aligned scaffolds and so both of these factors are likely to account for the differences in viability at day 12. In the second part of the study, cell number (assayed by total DNA) was higher at all time points across 21 days of culture on random fibres compared with aligned fibres most likely due to the higher porosity of the random scaffold facilitating greater scaffold infiltration and available surface area.

Fibrous scaffolds have been shown to promote cellular proliferation and differentiation because cells attach and organize around fibres with diameters smaller than their size [21]. In this study, scaffolds made of randomly orientated fibres generally caused fibroblasts to attach to multiple fibres whereas on aligned fibres they tended to spread along the length of fibres. On aligned scaffolds, cell orientation and migration could be precisely controlled by the direction of the fibres, a phenomenon called contact guidance theory [22]. However, at day 21 there were a small number of fibroblasts that deviated from the principle axis of alignment at the scaffold surface which may have been due to these cells experiencing space restriction and being unable to spread out along the fibre length as cell density has reached a critical point. It may be that these scaffolds need some form of mechanical strain, either statically fixed or dynamic, in order to maintain cell and collagen alignment throughout the whole construct after this time point. Mechanical conditioning has been shown to be effective for maintaining matrix alignment in fibrous constructs [16], [23].

It has been observed that fibre size can influence cell spreading [14], [24]. Previously, it has been shown that fibroblasts spread out more on microfibres (3.6 µm) compared with nanofibres (0.14–0.78 µm) [14]. An explanation for this is that focal adhesions, protein attachment complexes that connect the cell to the substrate, can be larger than 1 µm [24], [25] and so it is possible that submicron fibres undermine cell spreading by limiting focal adhesions size. In addition, the density of extracellular cell attachment proteins adsorbed to submicron fibres may be less than that on larger fibres. However, it is has also been suggested that cell expression of phenotypic markers is not achievable with the use of scaffolds containing fibre diameters that are equivalent to, or larger than the cell diameter [26]. Overall, it would seem that electrospun scaffolds need to be fabricated to suite the specific need of the cell type being used and tissue to be constructed with regards to fibre diameter, pore size, and fibre orientation.

Induced cellular orientation along well organized fibres has been reported in a number of cell types and it is believed that the various integrins are involved in this fibre-induced cell adhesion. Fibroblasts on flat PMMA substrates had vinculin (a protein involved in focal adhesion complexes) clustered around the cell periphery, however on nanofibres the integrin receptors were scattered across the entirety of the cell located everywhere the cell interacted with a fibre [27]. On microfibres, the integrin receptors were located along the edge of the fibre where the cell was adherent [27]. It has also been seen that human adipose-derived stem cells seeded on random fibrous mats possessed short and random vinculin plaques whereas cells on aligned fibres had long vinculin plaques orientated in the fibre direction [28]. These focal adhesions allow the cell to pull on the substrate and also pull on secreted matrix which may then result in organisation of this matrix. Fibroblasts were shown to orientate with deposited aligned collagen suggesting that they play a key role in collagen organisation but this requires further investigation.

Alterations in cell morphology can affect cell behaviour and gene expression [29]. Fibroblast alignment on highly orientated scaffolds appeared to result in a greater cell elongation than those seeded on random fibrous mats. It has been shown that elongation of the cell cytoskeleton also facilitates distortion and elongation of the cell nucleus, which affects cell differentiation and promotes DNA synthesis [29]. Although total collagen was equal on both scaffolds at all time points, when this was normalised to DNA, aligned scaffolds had more collagen per cell at day 21. Similar findings have been reported elsewhere, and it has been suggested that this may be due to denser collagen packing on aligned scaffolds due to the collagen fibres aligning with each other [16]. Matrix orientation, and more specifically, collagen orientation, dictates the mechanical properties of connective tissues. Non-invasive imaging cannot be achieved with many collagen visualisation techniques and other methods of quantifying collagen give no organisational information but it is important to monitor organisational development of collagen within the construct to avoid future mechanical failure. The novel technique of SHG, showed that collagen matrix appears highly orientated on aligned scaffolds and is retained throughout the depth, whereas fibrils appear to orientate in all directions on random scaffolds and change orientation throughout the depth of the scaffold. SHG intensity is dependent on a number of factors including, amount of collagen, fibre diameter, molecular organisation, and orientation [12]. SHG intensity was higher for all depths on aligned scaffolds than on random scaffolds. Sirius red was also used to visualise collagen deposition but this technique is destructive and non-specific, and fluorescent imaging showed that it does not offer a clear picture of how collagen fibrils are organised in the constructs.

It was also discovered that scaffold fibres could be visualised selectively via SHG dependent on the illumination wavelength which could offer a useful and novel method for monitoring scaffold degradation in future studies. SHG also proved useful for imaging fixed constructs, although signal was diminished due to collagen cross-linking. Finally, detailed 3D images of collagen organisation were collected which could aid with the creation of complex tissue models of healthy and diseased tissue. In future, with the use of non-descanned detectors, collagen SHG could be observed many hundreds of micro-meters into the construct, allowing for greater depth profiling.

Mechanical testing confirmed the influence that the matrix orientation had on the construct tensile properties. While all scaffolds became stronger with time, parallel fibres had the largest increases in *E* and UT as a result of the orientated collagen. The *E* of parallel stretched fibres was 250 times (day 14) and 100 times (day 21) greater than perpendicular stretched fibres. Tendons and ligaments have been shown to have tensile properties up to 500 times higher when measured parallel to the fibre direction compared to measurement in the perpendicular direction [30]. Randomly orientated scaffolds were about 10 times less stiff than parallel fibres at all time points, supporting the observation that collagen was organised in all directions as seen from the SHG images. Normal human skin has a tensile modulus of about 15–150 MPa [31] and while the randomly orientated constructs were not quite in this range at day 21, the aligned fibrous constructs were. It may be that a combination of random and aligned fibres may be used to better tailor the mechanical properties of tissue engineered constructs.

Pore size and shape varied between random and aligned scaffolds. Scaffolds with highly orientated fibres tend to have longer, narrower pores compared with randomly orientated mats due to a higher fibre packing density and the way the fibres align. The high packing density of the aligned fibres is required in order to offer structural integrity to the scaffold. Fibrous sheets produced by electrospinning are essentially layered in one plane and so continuously depositing fibres on top of each other results in dense fibre packing towards the middle of the scaffold. This causes pore size to decrease towards the centre of the scaffold due to increased overlapping of fibres compared with the surface, making it harder for cells to infiltrate and for the exchange of nutrients. However, there are many tissues in the body that have ECM organised in one plane includingtendon, dermis and the cornea, where the native fibrous network is similar to a series of layers stacked on top of each other. A strategy of layering up sheets of cell-seeded electrospun scaffolds to allow the construction of a thick 3D construct has been tested [32]. Using this technique, complex architectures can also be formed by layering a combination of different fibre orientations to mimic more complex tissues with depth dependent architectural organisations, for example cartilage [12]. It is also possible to increase scaffold porosity by co-spinning with a very fast degrading or water-soluble polymer e.g. PEG [33] or using mechanical techniques such as ultrasonication [34] to force apart fibres thus allowing greater cell penetration and nutrient diffusion. In contrast thin nanofibrous sheets can be electrospun between macroporous sheets to limit migration between two tissue types to create a multi-tissue [35]. A combination of these techniques may allow for the production of large 3D constructs with controllable porosity and fibre orientation.

## Conclusions

Cell behaviour can be controlled by altering the fibre arrangement of electrospun scaffolds, which has a direct effect on collagen production, organisation, and construct mechanical properties. The different fibre architectures that can be produced via electrospinning show promise in the production of scaffolds that mimic the native ECM of many different tissues including tendon/ligament (aligned fibres) and skin or cornea (random fibres). We have demonstrated that SHG can be an informative minimally-invasive tool for monitoring collagen production and organisation inside tissue engineered constructs to aid in the design and production of tissue-specific engineered matrices.

## Supporting Information

Figure S1 **PCL fibres and tendon SHG collected from 800 nm and 940 nm illumination.** SHG was observed emitting from both randomly organised and highly aligned PCL fibres using 800 nm illumination but there were no observable emissions from 940 nm. SHG was visualised from collagen in rat tail tendon at both illumination wavelengths.
(TIF)
