# Supplementary figures and images for: Monitoring Fibrous Scaffold Guidance of Three-Dimensional Collagen Organisation Using Minimally-Invasive Second Harmonic Generation
Source: PLoS One. 2014 Feb 28;9(2):e89761. doi: 10.1371/journal.pone.0089761 (PMC3938545; doi:10.1371/journal.pone.0089761)

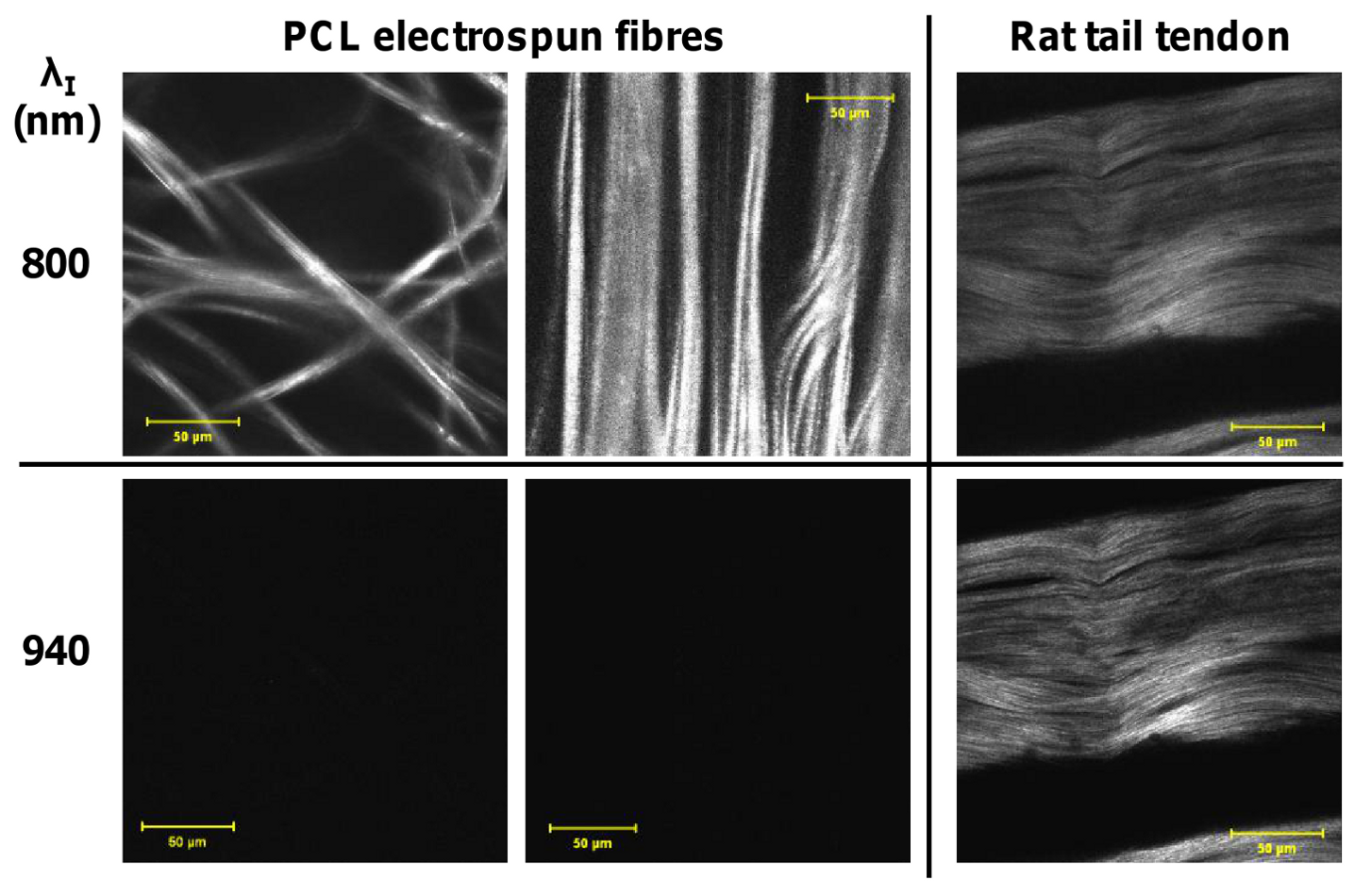

Supplement: Figure S1 — PCL fibres and tendon SHG collected from 800 nm and 940 nm illumination. SHG was observed emitting from both randomly organised and highly aligned PCL fibres using 800 nm illumination but there were no observable emissions from 940 nm. SHG was visualised from collagen in rat tail tendon at both illumination wavelengths. (TIF) [file pone.0089761.s001.tif]
